# Intelligent Design of Product Forms Based on Design Cognitive Dynamics and a Cobweb Structure

**DOI:** 10.1155/2021/6654717

**Published:** 2021-02-10

**Authors:** Wenjin Yang, Jian-Ning Su, Shutao Zhang, Kai Qiu, Xinxin Zhang

**Affiliations:** ^1^School of Mechanical and Electrical Engineering, Lanzhou University of Technology, Lanzhou 730050, China; ^2^School of Design Art, Lanzhou University of Technology, Lanzhou 730050, China; ^3^School of Architecture and Art Design, Hebei University of Technology, Tianjin 300401, China

## Abstract

Design is a complex, iterative, and innovative process. By traditional methods, it is difficult for designers to have an integral priori design experience to fully explore a wide range of design solutions. Therefore, refined intelligent design has become an important trend in design research. More powerful design thinking is needed in intelligent design process. Combining cognitive dynamics and a cobweb structure, an intelligent design method is proposed to formalize the innovative design process. The excavation of the dynamic mechanism of the product evolution process during product development is necessary to predict next-generation multi-image product forms from a larger design space. First, different design thinking stimulates the information source and is obtained by analyzing the designers' thinking process when designing and mining the dynamic mechanism behind it. Based on the nonlinear cognitive cobweb process proposed by Francisco and a natural cobweb structure, the product image cognitive cobweb model (PICCM) is constructed. Then, natural cobweb predation behavior is simulated using a stimulus information source to impact the PICCM. This process uses genetic algorithms to obtain numerous offspring forms, and the PICCM's mechanical properties are the energy loss parameters in the impact information. Furthermore, feasible solutions are selected from intelligent design sketches by the product artificial form evaluation system based on designers' cognition, and a new product image cognitive cobweb system is reconstructed. Finally, a case study demonstrates the efficiency and feasibility of the proposed approach.

## 1. Introduction

The development of new technologies brings major changes to the product form design process and subverts the traditional design mode, and the analysis of consumer demand and behavior becomes an important means for product design and marketing [1]. At the same time, the idea of intelligent design is intuitively reflected. The status quo of achieving innovation by only designers will be improved. However, design is a complex, iterative, and innovative process [2], and intelligent design systems based on algorithms are limited.

The genetic algorithm (GA) is an important method for intelligent design systems. Originally, GAs were used to find solutions to complex optimization problems in an evolutionary process [3]. Currently, this method has become a major means of exploring and evaluating design solutions, especially for two-dimensional (2D) sketches [4]. Hsiao used GAs to achieve the purpose of morphological innovation design [5]. Many studies suggest that the combination of design strategies with GAs guides the design direction [6–9]. However, some designers believe that intelligent design lacks the ability to discover a superior solution with extended boundaries but is limited to creating a local maximum [10]. Additionally, the industry also believes that intelligent design is difficult to widely use in real product development [11]. Therefore, it is important to integrate natural design cognition into intelligent design processes. The design cognitive dynamic mechanism and attention transferring mode have been described in an appropriate way and have been transformed into a computer language to form an advanced intelligent design method that supports the serialization, diversification, and individualized development of a product form. Designers always create sketches by a sudden inspiration. Similarly, intelligent design systems based on design cognition create innovative schemes in light of existing products and other novel forms [12]. In the concept generation stage, intelligent design systems generate a large number of visualized schemes to form a feasible solution space [13]. This space is a good theoretical foundation for practical product form design tools that offer a wider variety of solutions.

Currently, product system competition is fiercer than that of a single product, brand or industry from the past. With the rapid development of networks, traditional enterprises manage to survive, and emerging enterprises look for their place. These enterprises need to build a strong product system to resist the impact of other systems [14]. To supply niche markets, product seriation constitutes the product system, whose goal is to develop a modular platform and derive innovative products through a module configuration [15]. Numerous industrial examples have demonstrated the superiority of product seriation to ensure corporate competitiveness, such as Sony [16, 17] and Hewlett Packard [18]. Widespread recognition exists that platform-based development has a significant influence on the cost, performance, quality, and variety [19]. However, few studies have convincingly articulated these serialization strategies and applied them to the development of product systems.

Product seriation is an advanced form of standardization. Product seriation has two prongs: one is the identification of shared elements and the other is the generation of innovative forms based on shared elements. The purpose of identifying a shared element is to reduce manufacturing costs, while the purpose of generating innovative forms is to meet customers in various market segments by increasing competitiveness and differentiation [20]. A product genealogy diagram is formed with serialization, diversification, and individualized development. From a macroscopic point of view, the product genealogy diagram has a similar structure to a cobweb. The intersections of warp lines and weft lines, i.e., radial filaments and wire-harvesting, of the cobweb correspond to products. The warp lines correspond to the image series of the product. The weft lines correspond to the connection between product forms of the image series [21]. From a microscopic point of view, some significant characteristics of cognitive activity can be metaphorically reflected in such a nonlinear cognitive cobweb, and there is a nonlinear feedback loop between cognitive nodes [22]. In addition, Stefano Lenci believes that a cobweb can be used to reason the underlying cognitive dynamics at the microscopic level [23]. The spider silk in nature is a typical viscoelastic material, which creeps under the action of a force and has the appearance of a nonlinear stress-strain curve [24]. Therefore, we use the mechanical laws of a cobweb impacted by an external force to infer the cognitive law of designers in product seriation design, i.e., the nonlinear attenuation phenomenon of a product form image. We analogize the product system to the cobweb in nature. The force transmission of warp and weft lines is used to simulate the cognitive law of the potential image in the continuous process of product evolution design and series expansion.

In order to better understand the designer's cognitive model, and to establish effective computer-aided design intelligent systems, there is a need to construct characterization models of product seriation and to understand the impact of image information on the entire product series. Therefore, we propose a product seriation intelligent design method based on the designers' cognitive process and cobweb structure. First, a product image cobweb model is built by simulating the cobweb structure. Second, as a cobweb in nature receives an impact, the product image cobweb model receives information. Third, the model absorbs the information and outputs a new series of product forms using the GA. These new product form schemes are visualized by the cobweb model and are manually screened by designers. Therefore, using this model, we can continuously expand the product image series and predict the development trend of the product form. To the best of our knowledge, the evolution of product form series and the representation of the cobweb can make a positive contribution to computer-aided industrial design. We use GAs based on design cognition and cobweb mechanics to help designers deal with the complexity and uncertainty in the design process so that designers can explore complex design spaces and quickly produce diverse solutions. The representation of a cobweb better simulates the law of product seriation design. This study demonstrates that it is important to develop computational methods based on cognition to bring about the intelligent design of product seriation.

The remainder of this paper is organized as follows.  Section 2 presents the background and related studies of cognitive science, intelligent design, and cobweb theory. In Section 3, we introduce an intelligent design method of a product form based on the cognitive cobweb model.  Section 4 demonstrates the method through a case study of a household packaged air conditioner and highlights designers' cognition stimulation by external factors to achieve an intelligent design. In the end, we summarize the contribution of this study and future development directions.

## 2. Theory Background and Related Studies

### 2.1. Cognitive Science

Research in cognitive science and information processing is an important part of the International Human Frontier Science Program (HFSP). The Ulm School of Design introduced the scientific theory of general system theory, cybernetics, information theory, ergonomics and sociology, experimental psychology, mathematics, etc., which was just emerging at that time and laid the theoretical foundation for system thinking in the field of design and design science. Many scholars have also conducted a series of studies in this regard. By observing the design activities of architecture students, Lawson [25] summarized the cognitive style characteristics of the “emphasis on problems” and “emphasis schemes” and found that “emphasis schemes” are the designers' preferences in design problem solving. Gabriel et al. [26] proposed a design innovation model to address design problems. Liu and Sun [27] introduced a semantic representation of design intent to describe the design thinking process. At the same time, some intelligent algorithms were applied to the product concept design stage, such as the neural network algorithm, fuzzy set theory, and the GA. Kang and Tang [28] proposed a new method for analyzing conceptual design activities. These authors used similarity theory to analyze the compatibility of two adjacent components and automatically determine the appropriate design by an ant colony optimization (ACO) method. Liikkanen and Perttula [29] introduced memory-based creative generation to modify experiments in conceptual product design. Taura and Nagai [30] developed a systematic theory and found that the concept generation process can be divided into two categories: first-order concept generation and high-order concept generation. Chong et al. [31] adapted a general best first heuristic algorithm for computer-aided conceptual design (CACD) to strategically guide designers in exploring design solutions. In recent years, many scholars have studied cognitive science from the perspective of dynamic systems and proposed cognitive dynamic system theory. For example, Agmon and Beer [32] constructed a cognitive system by studying the interactions among the nervous system, human body and environment. The work explains the dynamics of perceptual classification. Springett et al. [33] established a human-computer interaction model by integrating emotional cognition with interaction and usability. Pollack [34] applied fractal and chaotic dynamics to construct a nonlinear cognitive dynamic system for identification and classification. Stepp et al. [35] constructed a cognitive dynamic model through a set of differential equations and described the transient phenomena of the system with multiple variables. Zednik [36] used the task parameters and evaluation criterion as inputs to construct a cognitive model with dynamic equations and to describe the interaction mechanism between the inputs. A study by Weiskopf [37] showed that composition and semantic interpretation constitute the causal structure of cognitive patterns and reveal the complexity of associations. Some scholars proposed a cognitive research framework based on cognitive computing and cognitive dynamic systems and believed that modern control theory is more suitable for constructing cognitive dynamic systems. Jin and Benami [38] developed a model for cognitive activity consisting of iterative loops in key cognitive activities, namely, analysis, generation, combination, and evaluation. Ma et al. [39] proposed a process for generating new ideas to explore the generation mechanism of solutions in conceptual design. Iarovyi et al. [40] studied contemporary solutions applicable for introduction of cognitive capabilities in manufacturing systems and proposed the architecture for cognitive manufacturing system employing benefits of industrial Internet and cognitive control. Gero and Kannengiesser [41] proposed a functional-behavior-structure (FBS) model. The FBS model was later modified by several researchers to be one of the most effective methods for expression and concept building. Image cognition is an important part of product design. Many scholars proposed different algorithms regarding cognitive matching of the nature of imagery vocabulary and established a variety of noumenon for product knowledge representation from different aspects [42], which was used to find the element matching pair between two ontology concepts [43].

### 2.2. Intelligent Design

GAs are the core of intelligent design. Rosenman [44] proposed a model of evolutionary design that became an effective tool for design. Lo et al. [9] constructed an evolutionary system for product forms utilizing GAs and aesthetic measurement principles to shorten the design process. Based on the concept of a product family, Bonev et al. [45] created an intelligent customized system to derive serialized product schemes. Lee and Chang [46] analyzed consumers' emotional responses to product form and proposed selection and evolution mechanisms to establish a product form design platform. Based on the existing research on biological evolution principles and GAs, Gero et al. [47–50] proposed a genetic simulation model in the design process. This process explained the possibility and necessity of intuitively applying the various phenomena of life sciences to innovative design. Tay and Gu [51] proposed a product evolutionary design method based on functional modules. Shieh et al. [52] established a product form design model based on multiobjective optimization and multicriteria decision-making. Haber et al. [53] presented a simple multiobjective cross-entropy method. Su et al. [54] used Kansei engineering to improve product image modeling and proved that the intelligent design method can effectively assist designers in designing the appearance of products. Lin and Wei [55] focused on consumers' affective preferences in relation to visual ergonomics to propose a new hybrid consumer-oriented model using gray relational analysis, gray prediction, and the technique for order preference by similarity to the ideal solution. On the basis of Kansei engineering, Guo et al. [56] selected variables of mobile phone design features by combining quantitative and qualitative analysis methods. A GA integrated with back propagation (BP) neural networks was used to optimize mobile phone solutions by considering design requirements and users' emotional needs.

### 2.3. Cobweb Theory

The cobweb-like structure is widely used in different disciplines because of its easy-to-understand structure and structured representation of information. The traditional cobweb model is a dynamic equilibrium model proposed in economics, where the price, supply, and demand of commodities change with time [57]. Therefore, through the analysis of the definition of the industrial chain, economists put forward the industrial chain cobweb model to improve profitability and efficiency. In the field of cognitive science, some scholars constructed a nonlinear feedback link between subjects and the world being cognized and described it by a nonlinear cognitive cobweb [22]. Additionally, the cognitive cobweb is continuously evolving [58]. Some scholars proved that the characteristics of the cobweb structure were mainly manifested for decomposing the powerful kinetic energy from the outside [59]. Based on the physical structure of the cobweb, Zhang et al. [21] simulated design thinking and proposed a product image cobweb model and algorithm.

Although the above studies have made significant contributions to product design, there are some drawbacks in systematic intelligent computer-aided design. First, few studies present the seriation of product families based on a systematic network structure. Second, it is necessary to conduct further research on how to clarify the direction of an intelligent design. Based on the design thinking process and natural cobweb structure, we propose an intelligent design method to guide the intelligent design of a product form with different images and construct a visualized evolutionary system. First, by analyzing the designers' thinking process to mine the dynamic mechanism behind it, stimulus information from different sources is determined. Second, during the conceptual design stage, the cobweb model and GA are introduced to construct a product form evolutionary system in line with design cognitive thinking. Third, the validity and feasibility of the proposed approach are verified by a case study. This paper purposes to realize the intelligent design of a product form by mining the dynamic mechanism in the design process and visualize the dynamic process of product development.

## 3. Methodology

### 3.1. Method Structure

The intelligent design method of a product form based on the cognitive cobweb model clearly characterizes the development of the product image form. The image cognitive cobweb model in our method is established based on image cognition and the relationship between the image lexical ontology models [60]. The angles between warp lines are determined by the correlation distance between image vocabularies. The positions of the weft lines are arranged evenly. Products are located at the intersections of the warp lines and weft lines according to their image value. The selection, crossover, and mutation operations of product forms are performed with intelligent algorithms. There are two important points in this operation. The first point is to give the parent sample a different stimulus. For example, when designers execute a design task, they need to give the image cognitive cobweb model a stimulus from the outside world so that the newly created product form exhibits a certain strong image. The second point is to determine the degree of influence of stimulus on the parent product according to mechanical properties of the cobweb, i.e.., to determine the corresponding crossover probability on each warp line, to reconstruct the product cobweb system.

**Figure 1:**
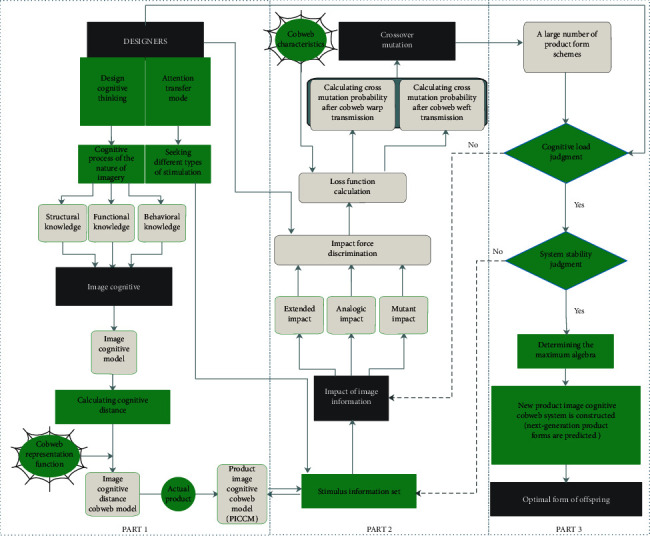
Method structure.

The methodological framework for the study is shown in Figure 1. The first step is to establish the product image cognitive cobweb model (PICCM). It is necessary to seek the imagery essence according to the designers' cognition and summarize the sources of stimulation from different image forms according to designers' attention transferring mode. On the basis of the concept of the nonlinear cognitive cobweb proposed by Francisco, this paper constructs a preliminary image cognitive model by combining the designers' knowledge of the structure, function, and behavior in product cognition. Furthermore, the position of warps is established by referring to the cognitive distance between image vocabularies. Thus, the image cognitive distance cobweb model is established. Finally, the corresponding products are located in the image cognitive distance cobweb model to form the PICCM. The second step is the impact of the PICCM. Information stimulation, i.e., impact is performed on the PICCM according to the stimulus obtained in the first step. The results of the impact are obtained by means of GAs. The GA used in this study is the bee evolutionary genetic algorithm (BEGA), which can introduce form genes in a reasonable form to achieve different degrees of impact. The mechanical properties of the PICCM are the energy loss in the impact information, which determines the degree of influence in the impact information on each intersection of the cobweb. The third step is the product form evaluation system based on the cognitive load of the designers. The process uses manual evaluation, and designers find the evolutionary algebra while looking for the solution from the intelligent design sketch. Finally, we reconstruct a PICCM, as shown in Figure 1.

### 3.2. PICCM

To more clearly visualize the dynamic process of product seriation development and to explore the dynamic factors that designers' cognition provides for the development of a product form, we construct a cobweb model based on designers' cognition. First, we analyze the cognitive thinking process of an image and find that people's expression of a product emotional image is subjective [61]. However, the vocabulary itself comes from the individual's cognition of certain imagery-related things. The vocabulary includes functional, structural, and behavioral knowledge and other knowledge regarding various products. Therefore, in this study, the cognitive distance between images is assumed to be equal to the semantic distance between vocabulary, and the distance between semantics is transformed into the image cognitive angle. Second, the biomimetic structure of the cobweb is used to characterize the product imagery, and the product of a certain brand is placed on different warp lines according to the image given by the designers. A Likert three-level scale is designed to visit several designers, and finally, the image value of the product is obtained. The position of the product is determined according to the size of the image value, and then the weft lines of the cobweb structure are determined, thereby establishing the final PICCM.

#### 3.2.1. Designers' Innovative Cognitive Thinking Process

From a cognitive point of view, product innovation design thinking is a brain intake of task information from the outside world, which is first transformed into a working memory and later extracted as previous design experience. The thinking process can stimulate the generation of a new memory, and then through the comprehensive treatment of human brains, design solutions are achieved. This is the reciprocating thinking process for solving problems with innovative solutions [62, 63]. From the perspective of the thinking mode, the product innovation cognitive thinking process includes both divergent thinking operations and agile thinking operations. The former is to explore the design information from the breadth to obtain more useful thinking directions; the latter is a deeper discussion of the evaluation, combination, and migration of the information obtained from the depth to enhance the pertinence and efficiency of the analysis problem.

In this paper, we mainly study two parts of design cognition. The first part is the designers' cognitive process of image vocabulary. Considering environmental factors and functional, structural, and behavioral knowledge as the main influencing factors of product design, the product image cognitive model is constructed. The second part is the designers' attention transfer mechanism. When the designer receives a certain design task, he will consciously seek certain stimulation information.

#### 3.2.2. Method for Establishing the Product Image Cognitive Cobweb System

Human cognition of imagery is expressed as a vocabulary, which is also the accumulation of people's constant understanding of things. The knowledge of the vocabulary is stored in the human brain, and similar to the information contained in a dictionary, this vocabulary stipulates the spelling or pronunciation of words and the meaning of words. In an ordinary dictionary, we use a known word to define a new word, and language is linked to the objective world through the interpretation of meaning. In human semantic memory, it is also necessary to express the relationship between the meaning and concept of a word, but its organization is different. The psychological representation of the meaning of a word is more complicated than the literal meaning of the word [64]. Through the study of lexical relevance at the beginning of this century and the study of psycholinguistics in recent years, a large number of research results have begun to reveal this complex lexical semantic relationship.

In this paper, we use the WordNet semantic dictionary to calculate the relevance of image adjectives. WordNet is a large English dictionary built and maintained by a Princeton University Science Laboratory under the direction of psychology professor George A. Miller. This dictionary has two advantages: the first is that it is easier to use than a simple dictionary; and the second is that it supports automatic text analysis and artificial intelligence applications. WordNet practices the idea based on the research results of psycholinguists, so it is called a dictionary based on the principles of psycholinguistics.

**Figure 2:**
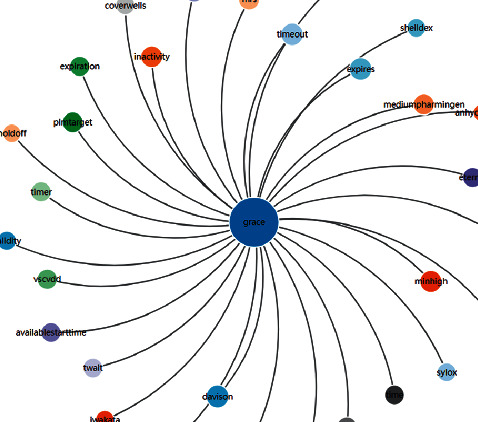
WordNet semantic analysis process.

The most important relationship discussed by WordNet is the synonymous relationship between lexical semantics because its ability to judge this relationship between words is a prerequisite for expressing meaning in a lexical matrix. The correlation analysis process of WordNet is shown in Figure 2. The image description vocabulary is limited to a specific range for the same type of product. For example, when describing a certain type of household appliance, we would say that it is gorgeous, luxurious, noble, etc., and we will not use strong, fierce, and other words that are usually used for electromechanical products. It has been proven that the performance based on the WordNet semantic distance algorithm is better than other algorithms in the field of image recognition [65]. Then, we built the cobweb structure according to the semantic network associated with the design [66]. The warps represent images, and there is a certain relationship between a warp and the other warps. Therefore, we convert the semantic relevance into an angle in the light of WordNet, and based on this, we initially construct the warp structure belonging to the cobweb; that is, the image distance cobweb model is constructed, and the correlation transformation formula is shown in equation (1).where *α*_*i*_ represents the angle between the images and *d*_*i*_ represents the semantic distance.

(1) $$\begin{array}{c} \alpha_{i}=\arccos\;d_{i}, \end{array}$$

First, after determining the research object, we collect the samples and search for the corresponding image entries behind each sample, extract keywords, and analyze them. Second, the final representative image vocabulary is analyzed with text deep mining. The corresponding vocabulary corresponds to different warps. Third, SD questionnaire analysis is performed on the products on different warps. A comprehensive analysis is conducted to obtain the average of the image cognition of designers and users, according to the size of the score in the line. The more developed the warp is to the periphery, the stronger the image represented by product is, and the higher the score is.

### 3.3. Method of Multi-Image Series Product Form Intelligent Design

We regard the serial design of a product family as a cobweb system. When certain elements in the system change, the entire system will change. This phenomenon is close to the change in the natural cobwebs after impact by external forces. Therefore, we named the intelligent design process based on the cobweb structure as the information impact and conduction process, and the impact force behind it is the designers' source of cognitive power. The principal part of the design activity is designer. Therefore, it is necessary to simulate the designers' attention transferring mode as much as possible in the intelligent design. In this study, the cognitive power source in the design process is used to simulate the computer; that is, we use the image source form applied by designers to impact the existing product and finally simulate the development of the entire product system.

#### 3.3.1. Product Form Intelligent Design under the Impact of Image Information

The cobwebs in nature are subject to changes in the overall structure during the predation process. Similarly, similar changes occur when the PICCM is impacted by external image information. Due to the particularity of the product's cobweb system, we assume that all impacts occur at intersection of the warp and weft lines. At each intersection, there is a product form with a certain image. When the corresponding information impacts the PICCM, it first intersects with the product form at the intersection. The impact point product retains the maximum external image information, and the product form of other imagery series is also subject to a certain degree of external image information due to the conductive properties of the spider silk. The magnitude of the impact level in this study is represented by the crossover probability between forms and the form in the evolutionary design. That is, the magnitude of the crossover probability represents the degrees of influence of the image information on the entire cobweb. After the impact, the cobweb design system first tends to be in an unbalanced state, and a series of new forms are produced in the process. Furthermore, due to the self-recovery of the cobweb system, it returns itself to equilibrium. This process allows new forms to be selected so that all newly generated forms in the whole system may have good image recognition. Through the study of the whole impact process, we excavate the dynamic factors that promote the evolution of the product and visualize the product evolution process by the cobweb structure to predict the progeny products when the PICCM is impacted by external information.

Artificial intelligence technology provides new theories and tools for product form innovation and brings new ideas in the development toward intelligent design. At the same time, the conceptual design process in the product form innovation is the result of the collaboration and interaction of multiple types from design participants. Therefore, the collaborative evaluation system based on cognitive science will develop into an important supporting part of product form innovation. With the development of technology, the future intelligent design of a product form will be more demand-oriented and knowledge-diversified. The product form intelligent design system should have a certain level of professionalism and high efficiency. This system can provide effective support for building product family systems, modular configuration design, and product family dynamic development models.

**Figure 3:**
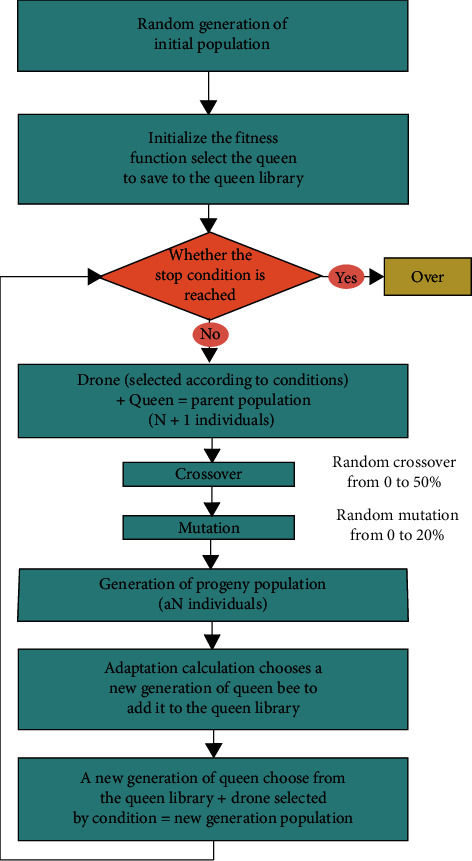
The genetic algorithm flow under the impact of image information.

The core technique of intelligent design is the GA, and the basic concept of the GA is to expand the initial population based on selection, crossover, and mutation mechanisms. Inspired by the way natural bees breed, the queen bee is always an optimal individual in the BEGA. The generated progeny inherits the best genes of the parents, so the evolution direction of the population becomes clear. At the same time, because BEGA introduces some random male bee individuals, new and individual characteristics are introduced on the basis of retaining good parental chromosomes, so the population has a wider evolutionary space [19]. Studies [67, 68] have proven that the BEGA can provide a method with good accuracy and speed convergence. These algorithmic features help us retain representative genes on the warp line of the cobweb. Therefore, we use the BEGA to generate a large number of forms for designers to choose in this paper. The BEGA flow under the impact of image information is shown in Figure 3.

#### 3.3.2. PICCM Parameter Calculation under the Impact of Image Information

(1) Balance of the cobweb system
The product system changes in the process of brand new product development, which is equivalent to a change in a cobweb in nature due to a certain impact force. The impact force needs to be controlled in a certain field. The cobweb will break if the impact force is excessively large, and if it is too small, it cannot cause a cobweb system change. Suppose that designers are given a special task; they generate a series of new ideas when they are stimulated by various types of information. The stimulus information cannot be too far or too close to the prototype. If the distance is too far, it will destroy the stability of the entire product system so that the entire brand loses its identity. If the distance is too close, it is difficult to meet the requirements of the new product development and design. Similarly, during the process of intelligent design, the degree of crossover between the stimulus information and product prototype should not be too high or too low. If the stability of the entire product system is too high, and if it is too low, then it is difficult to achieve a new product development that can satisfy the design requirements. That is, the degree of intrusion of these impact sources will be limited. All of the identification characteristics of the brand, the identification of the products, and the balance of the cobweb system must be guaranteed. In this study, we limit the range of the crossover probability from 0% to 50%.
(2) Loss function in image conduction
In Section 3.3.1, we mentioned that when a product at a certain intersection is impacted by a certain force, the surrounding intersection will be affected to some extent. Considering the product design system as a cobweb, the mechanical properties of the spider silk itself and the distance between the cobweb nodes directly affect the energy conduction process and determine the degree of energy loss. Therefore, this study uses the stress-strain characteristics of the spider silk to explain the loss process of image information during the conduction process.
 Excellent tensile mechanical properties are the main characteristics of spider silk. Some scholars have systematically studied and analyzed the tensile mechanical properties and formation mechanism of spider silk [24]. Based on the relationship among the morphological structure, aggregate structure, and the mechanical properties of the spider silk, the stress-strain behavior of the spider silk is simulated by the combination of a spring and glue pot, and a mechanical model of the tensile deformation behavior of the spider silk is established. The spider silk has a poor metastable structure, and once it loses its tension on the net, it will immediately relax and shorten its length. When an external force is applied again, the molecular chain inside the fiber may undergo morphological changes and rearrangement. After an elongation of approximately 30%, the stress-strain behavior of the fiber returns to the state of no relaxation.
As the external force increases, the fiber gradually forms a slow elastic deformation, and the stress-strain relationship at this time tends to be complicated. Under a certain external force, the basic stress-strain relationship in the cobweb mechanics model is
  (2) $$\begin{array}{c} \varepsilon_{i}=\frac{\sigma }{E_{i}}\left(1-e^{\left(\left(-t\right)|/|\tau \right)}\right), \end{array}$$
 where *σ* represents a constant and *E*_*i*_ represents the form variable of the simulated spring during the process of stress, and it is the only parameter for solving the elastic modulus of the spring.
 In the process of solving the energy loss of the PICCM, we assume that the elastic modulus of the simulated spring is certain and that the energy loss process of the spider silk indirectly reflects the energy loss process. The expression of the energy loss is
  (3) $$\begin{array}{c} \mu =1-e^{-\left(d/\tau \right)}, \end{array}$$
 where *μ* represents the amount of the loss probability of the crossover, *d* represents the distance from the point of the impact to the point of the solution, and *τ* represents the relevant mechanical parameter at the point of the impact of the cobweb.
(3) Morphological assessment method
 The intelligent design process is also a preferred process. The constant selection of new product forms is also a key factor in the continuous evolution of product populations. The design process is actually a subjective, emotional process. Therefore, in the research process of this paper, the method of manual evaluation is used to assist selection. The process is based on the aesthetics of product designers and their own awareness of the image of the product. The final selected form meets the requirements of two aspects: one is to meet the task requirements, and the other is that it cannot affect the designers' perception of the image. Then, the designer makes an in-depth design of the selected samples.

## 4. Case Study

To demonstrate the usefulness of our method, we apply it to the form design of a household packaged air conditioner. There are different series in this product. Product design sources and designers' design inspiration sources are collected through the product introduction page, and a text library is created. According to the results from using the text mining tool, frequent words and their frequency are identified. This research extracted the adjectives, which express the household packaged air conditioner, and a total of seven key images are mined: grace, honorable, gorgeous, smart, natural, fresh, and romantic. Then, the cobweb model is established accordingly. A total of 33 packaged air conditioning samples meeting the requirements were collected in the case study. Excluding too closed samples, a total of 20 samples were obtained. The results are shown in Table 1.

### 4.1. Cobweb Model Based on the Designers' Cognition of a Packaged Air Conditioner

#### 4.1.1. Constructing the Warp Structure

We look for the relationship between one another in the acquired image and use the WordNet semantic dictionary to calculate the semantic distance between each word meaning. The results are shown in Table 2. First, we count the word frequency of all sample implied images and obtain the highest frequency vocabulary as the main image vocabulary to determine its location. In this case, the vocabulary of the subject obtained was “honorable”.

**Figure 4:**
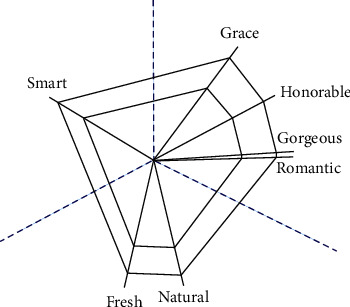
Image distance network based on the cobweb warp structure.

To construct a system model based on the cobweb warp structure, the images are first divided into three groups to exclude the complex relationship among images. One group is closer to the main image, including honorable, grace, gorgeous, and romantic. Another group includes fresh and natural. Another group includes smart. According to equation (1), the preliminary warp structure of the cobweb is constructed, as shown in Figure 4. In the figure, the blue dotted line is the dividing line of the three sets of image adjectives.

(4) $$\begin{array}{c} \beta_{i}=\frac{2\pi }{\sum_{i=1}^{n}\alpha_{i}}\alpha_{i}. \end{array}$$

**Figure 5:**
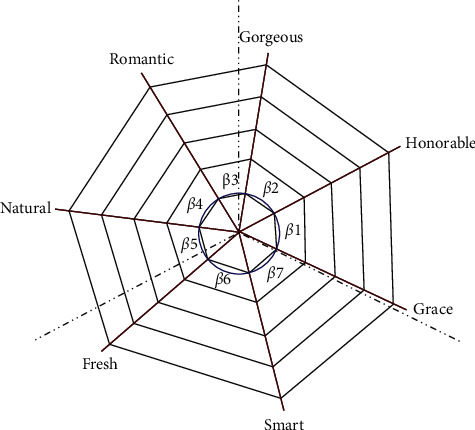
Positional relationship of the warp and weft.

By transforming the semantic relevance into the angle between the two image warp lines, the order is adjusted according to word correlation, and the data are normalized so that the sum of all angles is 360°. The conversion method is represented by equation (4). The results are shown in Figure 5, where *β*_1_ = 53.1°, *β*_2_ = 52.7°, *β*_3_ = 40.8°, *β*_4_ = 51.36°, *β*_5_ = 48°, *β*_6_ = 63.3°, and *β*_7_ = 50.74°.

#### 4.1.2. Constructing the Weft Structure

In the intelligent design of the cobweb system of the packaged air conditioner, the air conditioners with different images are arranged on different warps. The order is arranged according to the strength of the image of the product itself. The image is gradually enhanced as the warp extends outward. We assume that the product image is uniformly incremented on the warp and the weft structure of the cobweb system is constructed, as shown in Figure 5.

#### 4.1.3. Constructing the Cobweb Model across Images

Based on the cobweb structure constructed above, the image value relationship between each sample under the same image is found, and the product form is placed in the corresponding position to obtain the image cognitive cobweb system for the packaged air conditioner. In this process, we use the Likert three-level scale to score the image value of the different samples. A total of 50 questionnaires were distributed, and 46 valid questionnaires were returned. There were 20 women and 26 men. Twenty-three people are engaged in design-related occupations, and 23 people are engaged in occupations unrelated to design. Most participants were between the ages of 18 and 35 years.

The results are shown in Table 3 after the statistical analysis of the survey data.

**Figure 6:**
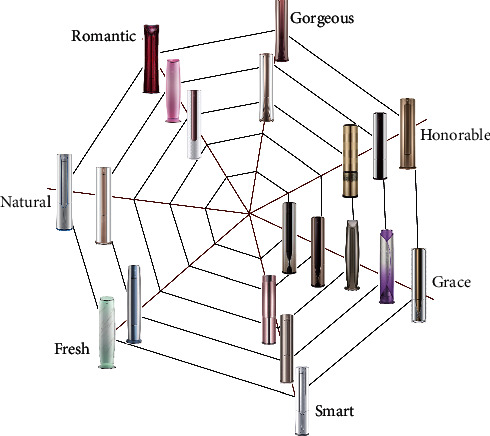
Household packaged air conditioner imagery cognitive cobweb model.

We place each imagery air conditioner pattern on the corresponding intersection of the warp and weft lines according to the following rule: if the warp is further developed to the periphery, then the image being transmitted is stronger, as shown in Figure 6.

### 4.2. Intelligent Design Method of the Air Conditioner Form Based on the Cobweb Impact

**Figure 7:**
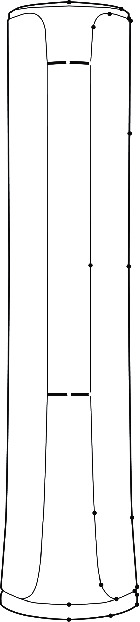
Parameterized sample 10.

First, the sample is parameterized. The construction of either complex or simple forms must be based on the key contours in the form design process. The process of expressing the form is to summarize the line by points and summarize the surface by lines. The essence of the expression process is to describe key control points of the contour line. This study describes the product form by the principle of the B-spline curve [69]. In the case of inconsistent constraints, the method can be processed and can effectively describe the form, and it is widely used in the form description process. Taking sample 10 as an example, the curve is parameterized, and the result is shown in Figure 7. Then, the sample is encoded. As a key point, the line or face of a form is used as a variable, and each component is defined on a finite interval. The size of the interval is determined by the type of the expressed gene. As shown in Figure 7, the air conditioner samples are coded with key point coordinates as variables. All the strings of the gene are superimposed, and finally its gene expression is determined. A set of strings consisting of 0 and 1 is obtained, and it is the chromosome.

**Figure 8:**
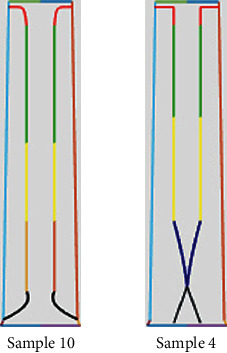
The impacted product and the impact product.

**Figure 9:**
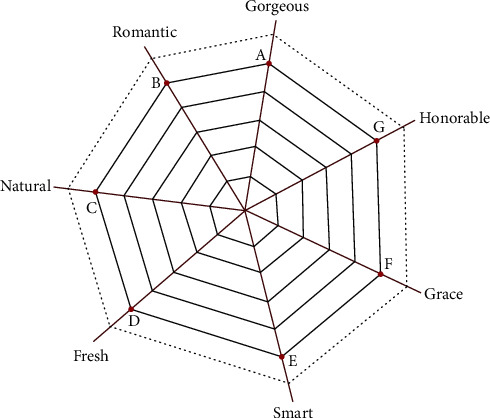
Reconstructed cobweb structure.

According to the needs of customers in the actual development process, we need to integrate the “grace image” into the entire air conditioner product system. Then, we explore the source of the cognitive power of the designers based on this assignment and found that designers often refer to the information in the same field—“grace air conditioning” to expand the design. According to the representation of the cobweb, the impact product we chose is sample 4 with a “grace” image. Designers believe that a gorgeous and grace air conditioner should be developed at first according to actual situation of the brand. Thus, the impacted product that has been chosen is sample 10 under the “gorgeous” imagery. The impacted product (sample 10) and the impact product (sample 4) are shown in Figure 8. The impact point is point A in Figure 9. It is assumed that a complete crossover operation occurs at the impact point; that is, the crossing probability at point A is 0.5 according to the loss function in the image conduction process and the balance of the cobweb system.

### 4.3. Cobweb Model after Reconstruction

We have stated in the above theoretical system that when the cobweb system is impacted, the surrounding points will experience some fluctuations, and the degree of fluctuation is determined by the performance of the spider silk and the distance between the intersection and the intersection point in the cobweb. The greater the distance is, the greater the loss is, and the less the product form is affected. In the packaged air conditioner image cognitive cobweb model, a new generation of products is produced and a new cobweb system is reconstructed, as shown in Figure 9. According to equation (3), the loss amount *μ*_*i*_ of points A, B, C, D, E, F, and G and the degree of influence *p*_*i*_ (crossover probability) of the point can be obtained, as shown in Table 4, where *d*_*A*_ = 0, *d*_*B*_ = *d*_*AB*_, *d*_*C*_ = *d*_*AB*_ + *d*_*BC*_, *d*_*D*_ = *d*_*AB*_ + *d*_*BC*_ + *d*_*CD*_, *d*_*E*_ = *d*_*AG*_ + *d*_*GF*_ + *d*_*FE*_, *d*_*F*_ = *d*_*AG*_ + *d*_*GF*_, and *d*_*G*_ = *d*_*AG*_.

**Figure 10:**
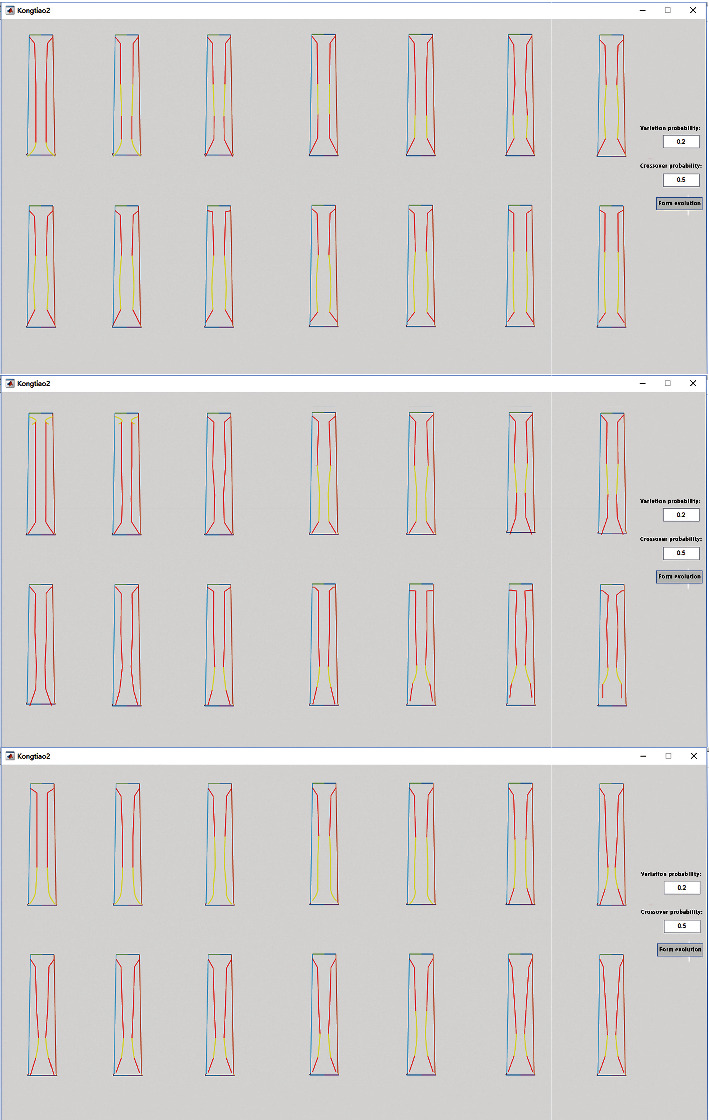
Some of evolutionary results in the impact point A.

**Figure 11:**
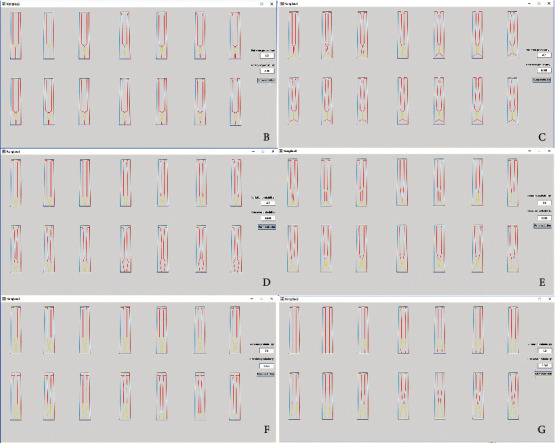
Some of the evolutionary results in the remaining points.

Different evolutionary results at different intersections by different crossover probabilities are produced on the warp of different images, and some of the evolutionary results of the impact point A are shown in Figure 10. Some of the evolution results from the impact of the impact force to the remaining points are shown in Figure 11.

**Figure 12:**
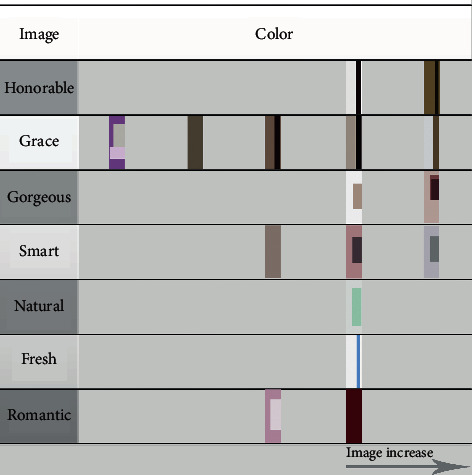
Image color network.

After the evolution process, the number of new schemes at all intersections is greater than 50. These schemes are then selected by designers to obtain the one that best meets the needs of the design image. Through the color extraction system proposed by Liu et al. [70], we extracted the corresponding colors of each product. Then, a corresponding color image network is established, as shown in Figure 12.

**Figure 13:**
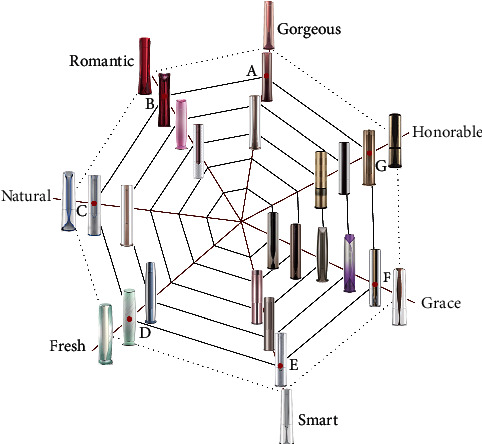
Reconfigured air conditioner cobweb system.

According to the color image network, a series of detailed processing steps, such as modeling and rendering of the selected sketch form, are performed to reconstruct a new cobweb that is shown in Figure 13. From Figure 13, the outermost circle of the cobweb is the predicted next-generation product form with a cross-image. Based on this product form, the designers believe that the cobweb model based on design cognition may output rich design knowledge, which greatly helps the design of the product. In addition, the intelligent design method based on cobweb impact is considered to be available through the evaluation results of designers.

## 5. Discussion

**Figure 14:**
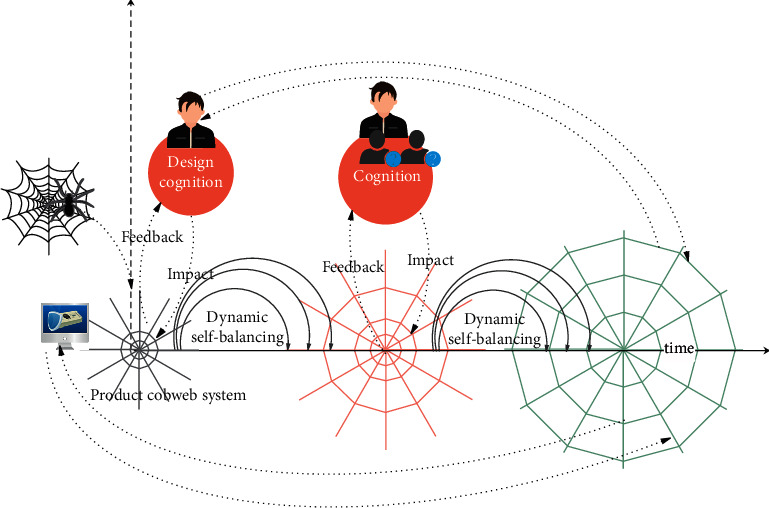
The model structure of the entire instance system.

In summary, the system established in this study is mainly divided into two parts. One part is the construction of the PICCM, and the other is the impact on the PICCM. The first part of the work focuses on analyzing the designer's cognitive thinking and obtaining the cognitive nature of the image. The images behind the serial design are excavated, and sources of stimuli for different types of designs are sought out during the process. The warp of the cobweb is established by calculating the semantic similarity of the image, and the weft of the cobweb is determined according to different product evaluations, thereby forming a PICCM. The second part of this study focuses on the use of GAs to achieve the impact of different stimulus information. In this process, the corresponding parameters of the GA are obtained by simulating the mechanical properties of the spider silk. Then, a large number of offspring forms are obtained. Therefore, a new cobweb model is reconstructed, and the next generation of product forms is obtained. The model structure of the entire system is shown in Figure 14. In Figure 14, we believe that the product cobweb model is constantly evolving and expanding under the cognitive driving force. The knowledge of designers and users continues to stimulate the product cobweb system. At the same time, the product cobweb system will give feedback to the designers and users, and the two have mutually reinforcing effects.

Effectiveness and constructiveness exist in any cognitive activity. Spiders interrelate with the external environment through the destruction and reconstruction of the network [23]. According to the nonlinear cognitive cobweb model proposed by Francisco [22], an intelligent design method is proposed by following the designers' thinking process. This method creates different intelligent designs by giving the PICCM stimuli according to the process of simulating the designers' inspiration during the designers' cognitive process. We consider an entire product system as a cobweb model. We believe that the product system is a process of continuous development and constant self-balancing over time because the natural cobweb has a strong adaptive ability. Therefore, the product intelligent design system based on design cognition and the cobweb model is actually a reconstruction process that is continuously extended and self-balanced due to the impact. In this process, the designers' knowledge and experience continue to impact the cobweb system, and increasingly more characters, such as users and engineers, will participate in the impact process with the development of intelligent manufacturing technology. We believe that intelligent design-generated solutions can meet a variety of user needs and are more in line with the actual production process. At the same time, the product cobweb system constantly feeds back new knowledge and experience to designers and computers throughout the development process.

We believe that the stimulating source of the impact brings a constant source of power to the product cobweb system by imitating the cobweb characteristics in nature. This process is in a state of dynamic balance, and the impact promotes the development of the entire system. In the example verification, we calculate the cross-probability loss by means of the mechanical properties of spider silk. Different impact positions and different sources of stimulation will generate a large number of corresponding new generation schemes. The final extensive sketch space provides designers with more feasible solutions. The refinement of the deep-design solution was recognized by relevant enterprise designers, which proves the reliability and effectiveness of the system to a certain extent.

The purpose of this paper is to propose a new approach, so some aspects of the case study may seem incomplete, which may be improved in future research. Certainly, our work has some limitations that provide opportunities for future research. We invited six designers to discuss the method. The discussion mainly focuses on two aspects: the availability of the method in the design industry and the adaptability of the method in the design process. Each designer participating in the interview agreed that the proposed method could be used in design practice. Interestingly, all designers believed that the representation of nonlinear cognitive cobwebs and the sketching schemes derived from intelligent design based on design cognitive dynamics are critical to their design process. Designers are often asked to design products that are similar to their previous designs or competitors' designs. The characterization of prior knowledge by the cobweb is very important for them in this process. One interviewed designer said: “Customers always bring similar products on the market when communicating design direction. We always refer to similar designs when designing to better understand the design direction.” Therefore, the source of the stimulus behind the mining design task is critical in the design process and will directly impact the design results. Intelligent design based on design cognitive dynamics adapts to the entire development process. Every designer involved in the discussion said that there are two major difficulties when designing the appearance of the product. First, it is difficult to meet the needs of customers because customers will heuristically judge whether the design is the same as what they want. Second, designers must consider brand constraints, but the appearance of the product will always change when it meets brand constraints. Designers said they not only better communicate with customers but also design more effectively by providing a large number of solutions with quantitative value for the product appearance.

However, the designer noted that the visual quality of the solution provided by the intelligent design based on design cognitive power needs to be improved. The designers believed that color, material, and surface technology are another important focus with respect to product expression. In this study, we focus on the process of constructing and reconstructing the PICCM of form. How color, material, and surface technology are directly expressed during the process of intelligent design will be carried out in subsequent studies. We will develop a method for converting 2D data into renderings through machine learning. This future work will help solve the problem.

## 6. Conclusions

We introduced a new method for the innovative design of imagery in a series of products based on a nonlinear cognitive cobweb model. The method is adaptive and sustainable. First, we combined a natural cobweb structure and the nonlinear cognitive cobweb model proposed by Francisco. The cobweb model structure is more suitable for serialized expression than other network structures. Therefore, we relied on the cognitive cobweb model to construct a static cobweb network system that can express the development of serialized products. Second, prey is the root cause of the evolution of an actual cobweb. Learning from the phenomenon of how spiders interrelate with the external environment through the destruction and reconstruction of the network, we determined the cognitive dynamics of the designer behind the design task as a source of stimuli that impacts the PICCM. The dynamic balance in the innovation and development of the product system is simulated through a series of strain reactions that occur when a natural cobweb is hit by prey. We completed the prediction of the next generation of products in the end. A new product system is produced based on the impact of a certain node. This method is a good illustration of the process of creating new solutions in the designer's design process and the process of influencing other product series forms in the system. Third, this study provides the basis for the computational generation of the design. The introduction of GAs makes the sketch generation process faster and offers more possibilities for designers. We used the innovative design of a series of household packaged air conditioners as a case study to verify our approach, focusing on the process of product serialization characterization based on the nonlinear cognitive cobweb model and the process of cobweb reconstruction after the impact of stimuli on the PICCM.

To ensure the versatility of the constructed cobweb model, we use the method of text mining to determine product image vocabulary and then use a natural language processing method to transform the semantic distance of the vocabulary into an angle to construct the warp of the PICCM. Designers can apply our approach to the serial design of products. Unlike existing innovative design methods, our approach characterizes the dynamic development of the product family system in a more intuitive way. An intelligent design process that integrates cognitive science and design thinking can help designers address the uncertainties in the design process. This process can quickly solve practical problems during the process of developing products. Designers can make decisions by using their specific area of expertise, making the design process more practical and widely used by enterprises. In fact, the entire product network will change after the cobweb system is impacted. The product form of the new generation will also have an impact on the original product form. There will be future research regarding this influence. Another area of research that we have discovered in our research is the process of incorporating irrelevant stimulus information into intelligent design. This process requires further research on the stability of the PICCM, which will be a future research direction. In addition, we are developing a method of converting 2D data into renderings through machine learning based on the above discussion, and we will consider building a cloud-based method architecture [71]. This future study will be beneficial for many industries, such as product design, apparel design, furniture design, and other fields.

## Figures and Tables

**Table 1:** Results of image value statistics.

Samples	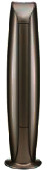	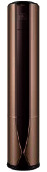	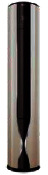	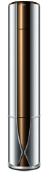	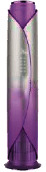	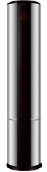	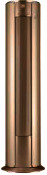	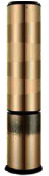	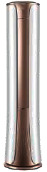	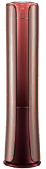
No.	1	2	3	4	5	6	7	8	9	10
Samples	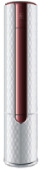	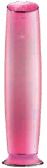	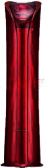	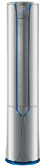	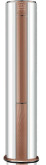	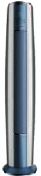	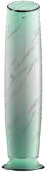	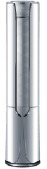	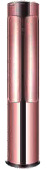	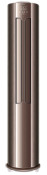
No.	11	12	13	14	15	16	17	18	19	20

**Table 2:** Seed collection location and date of the eight study species.

	Honorable	Grace	Gorgeous	Smart	Natural	Fresh	Romantic
Honorable	1	—	—	—	—	—	—
Grace	0.026	1	—	—	—	—	—
Gorgeous	0.037	−0.012	1	—	—	—	—
Smart	−0.066	0.014	0.123	1	—	—	—
Natural	−0.137	−0.035	0.058	−0.083	1	—	—
Fresh	−0.072	0.205	−0.086	−0.027	0.174	1	—
Romantic	−0.010	0.116	0.374	0.093	0.077	−0.029	1

**Table 3:** Results of the image value statistics.

Image type	No.	Comprehensive rating
Grace	1	2.20
	2	2.04
	3	2.02
	4	2.28
	5	2.26

Honorable	6	2.09
	7	2.35
	8	1.80

Gorgeous	9	2.24
	10	2.26

Romantic	11	1.91
	12	2.04
	13	2.11

Natural	14	2.35
	15	1.98

Fresh	16	2.07
	17	2.26

Smart	18	2.11
	19	1.74
	20	1.85

**Table 4:** Loss calculation result.

	A	B	C	D	E	F	G
*d*_*i*_	0	3.49	7.82	11.89	13.4	8.9	4.44
*μ*_*i*_	0	0.25	0.395	0.455	0.465	0.415	0.295
*p*_*i*_	0.5	0.25	0.105	0.045	0.035	0.085	0.205

## Data Availability

The data used to support the findings of this study are included within the article.
